# Teaching the Blind to Find Their Way by Playing Video Games

**DOI:** 10.1371/journal.pone.0044958

**Published:** 2012-09-19

**Authors:** Lotfi B. Merabet, Erin C. Connors, Mark A. Halko, Jaime Sánchez

**Affiliations:** 1 Department of Ophthalmology, Massachusetts Eye and Ear Infirmary, Harvard Medical School, Boston, Massachusetts, United States of America; 2 Berenson-Allen Center for Noninvasive Brain Stimulation, Department of Neurology Beth Israel Deaconess Medical Center, Harvard Medical School, Boston, Massachusetts, United States of America; 3 Department of Computer Science and Center for Advanced Research in Education (CARE), University of Chile, Santiago, Chile; University of Montreal, Canada

## Abstract

Computer based video games are receiving great interest as a means to learn and acquire new skills. As a novel approach to teaching navigation skills in the blind, we have developed Audio-based Environment Simulator (AbES); a virtual reality environment set within the context of a video game metaphor. Despite the fact that participants were naïve to the overall purpose of the software, we found that early blind users were able to acquire relevant information regarding the spatial layout of a previously unfamiliar building using audio based cues alone. This was confirmed by a series of behavioral performance tests designed to assess the transfer of acquired spatial information to a large-scale, real-world indoor navigation task. Furthermore, learning the spatial layout through a goal directed gaming strategy allowed for the mental manipulation of spatial information as evidenced by enhanced navigation performance when compared to an explicit route learning strategy. We conclude that the immersive and highly interactive nature of the software greatly engages the blind user to actively explore the virtual environment. This in turn generates an accurate sense of a large-scale three-dimensional space and facilitates the learning and transfer of navigation skills to the physical world.

## Introduction

Considerable interest has arisen regarding the educative potential of computer based video games and the behavioral and neurological effects associated with game play [1]. In particular, it has been suggested that the open structure and free discovery of information inherent in game based virtual reality environments improves contextual learning and the transfer of situational knowledge and awareness [2], [3]. Successfully leveraging these advantages in education and rehabilitation arenas has immense appeal and could potentially facilitate the learning of demanding tasks and further promote the transfer of acquired skills beyond the limitations of the training context itself [4], [5].

One interesting application of a video game based learning strategy would be to assist in the education and rehabilitation of individuals with profound visual impairment. For example, in the blind, navigating effectively is a very difficult task to master. Unlike sighted, blind individuals must rely on other sensory channels (such as hearing, touch, and proprioception) to gather relevant spatial information for orientating, route planning and path execution [6]. The mental representation of surrounding space is referred to as a “spatial cognitive map” [7]). Given the important role visual cues play in navigating, it has been assumed that blind individuals (and in particular, those who are congenitally blind) would be unable to create accurate mental spatial representations of their surroundings [8], [9] (see [10] for further discussion). Based upon immediate perceptual experiences in their close vicinity, this mental representation would be largely egocentric or “route” based. As such, this level of spatial organization would fail to capture the more global or holistic interrelations between objects in the surrounding environment. It would follow that blind individuals would be particularly challenged in situations when faced with large-scale unfamiliar environments or when alternate routes need to be taken [11]. Indeed, navigating effectively requires the ability to mentally manipulate spatial and contextual information, and for the blind, developing high-level spatial skills (related to allocentric-based or “survey” knowledge) is considered crucial for promoting greater travel independence [10], [12].

We hypothesized that these observed navigation difficulties reflect more the inadequate access of crucial and contextually related spatial information needed to characterize a given surrounding environment rather than holding the presumptive view that the blind have inherently impaired mental spatial constructs. Put another way, we wondered if a novel learning approach employed for capturing and understanding crucial spatial relationships of a given particular environment could influence overall navigation performance and the transference of skills.

**Figure 1:**
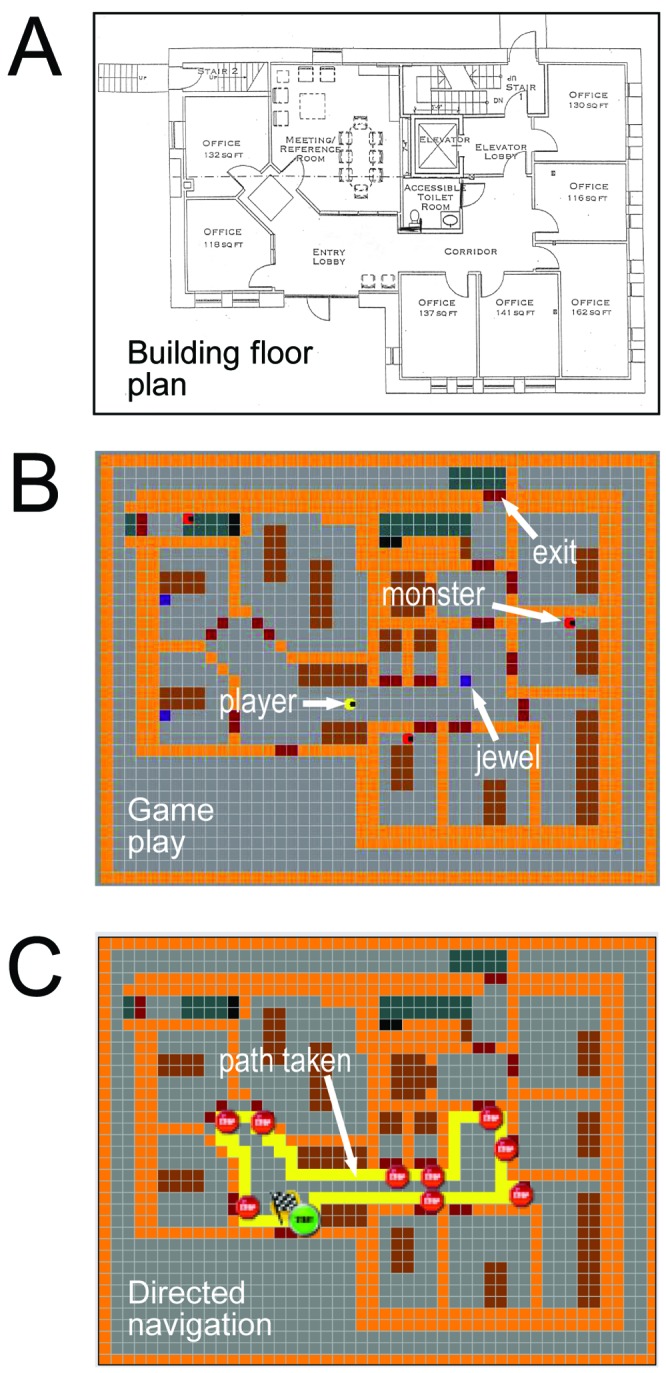
Virtual rendering of a physical environment represented in the AbES software. A) architectural floor plan of an existing two story building with 23 rooms, 2 stairwells and 3 exits. For simplicity, only the first floor is shown. B) In gamer mode, the player (yellow icon) navigates through the virtual environment using auditory cues to locate hidden jewels (blue squares) and avoid being caught by chasing monsters (red icons). C) In directed navigation mode, the user learns the spatial layout of the building and the relative location of the rooms using predetermined paths (shown in yellow) and with the assistance of a facilitator.

With these strategies in mind, we developed a virtual environment simulator training platform for the specific purpose of enhancing way finding skills in the blind. Audio-based Environment Simulator (AbES) is a novel, user-centered virtual environment that allows for simulated navigation and exploration of the layout of an existing physical building and set within an action video game metaphor (Figure 1. See methods for software description). We hypothesized that interacting with AbES would not only allow a blind user to generate an accurate spatial cognitive map of a target building, but also allow for the transfer of acquired spatial information to a large-scale, real-world indoor navigation task. Finally, we hypothesized that acquiring spatial information through the context of playing an action video game (as compared to an explicit route learning strategy) would promote improved contextual learning and situational knowledge demonstrable as enhanced navigation performance.

In this study, early blind participants (not previously familiar with the spatial layout of the target building and naïve to the overall purpose of the investigation) were randomized to one of two groups. In the “gamer” group, participants interacted with AbES within the context of a goal directed action video game designed to promote full exploration of the virtual environment. The game’s premise is to navigate and explore the entire virtual building so as to collect jewels (hidden in various rooms) while avoiding roving monsters that can take the jewels away and hide them elsewhere in the building (Figure 1 B). The gaming participants were encouraged to collect as many jewels as possible. By comparison, participants relegated to the second “directed navigator” group were explicitly taught the spatial layout of the building using AbES through a series of pre-determined paths with the assistance of a sighted facilitator. The training involved a complete step-by-step instruction of the building layout such that all the room locations, exits, and landmarks were encountered in a serial fashion (following a clockwise direction) similar to a “shoreline” strategy along the interior perimeter. The paths followed were virtual recreations of a typical lesson taught by a professional orientation and mobility (O&M) instructor for the blind (Figure 1C).

Following the training period, participants in both groups were taken to the target physical building to partake in a series of behavioral navigation tasks. In the first experiment, participants were instructed to navigate a series of predetermined paths. The paths were a series of start and stop points (i.e. rooms) whose pairing and sequence was unrelated to the explicit virtual training paths used to teach the building layout (i.e. in the directed navigation group). Primary outcome measures included whether the participant was able to successfully complete the navigation task and time taken to target. In a second experiment, a series of “drop off” tasks were carried out in which participants were placed at predetermined locations and instructed to exit the building using the shortest path possible (i.e. choosing one from three possible exits). Again, the possible paths used to exit the building from these start points were unrelated to the explicit virtual training routes and the paths tested in experiment 1. Performance in the latter task was scored for the path chosen (see methods section for scoring strategy).

## Results

### Experiment 1

**Figure 2:**
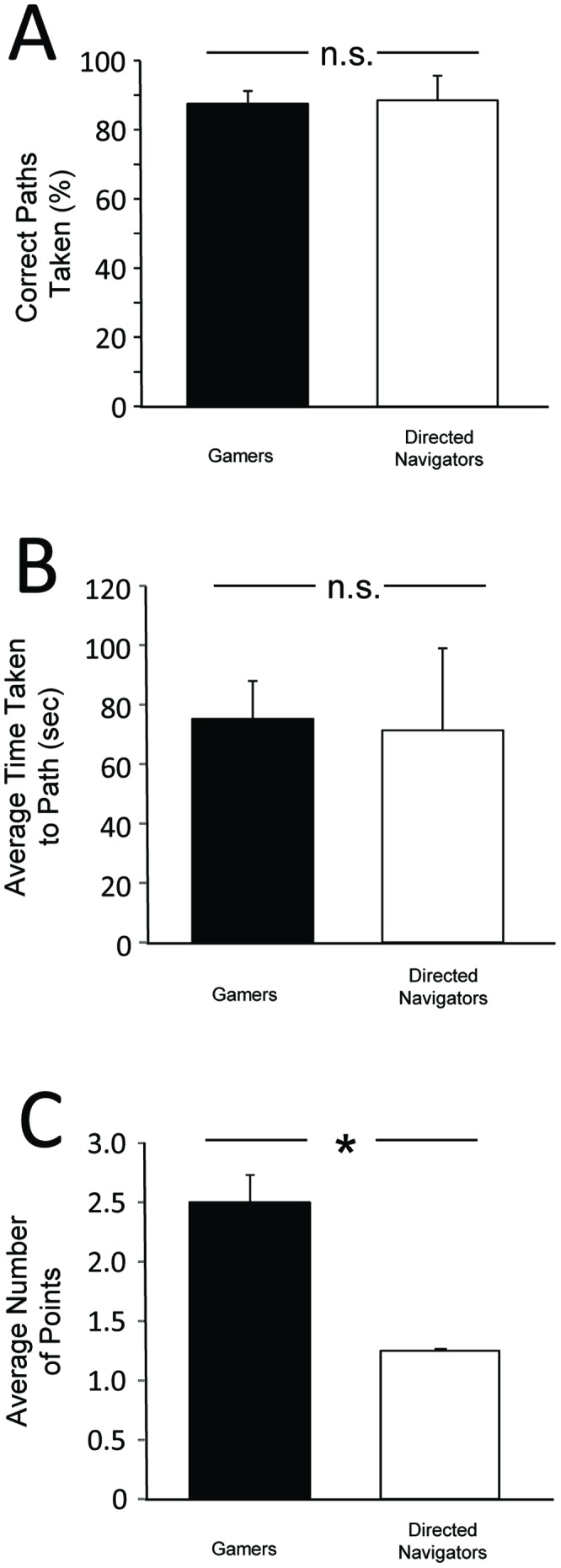
Comparing performance on navigation tasks between gamers and directed navigator learning strategy. A) High success on correct paths taken (%) was observed in both groups for room-to-room navigation. B) Average time taken to navigate to target (sec) was also similar in both groups. C) Results of drop off task reveals an advantage for gamers. Paths chosen were scored such that the shortest route possible to exit the building from a given starting point received a maximum of 3 points, 2 for next closest exit, 1 for the longest, 0 for unsuccessful). Gamers showed an advantage over directed navigators in that they were more likely to choose the shortest path on the drop off task (indicated by higher average point score). Error bars indicate S.E.M., n.s.  =  not significant. * = p<0.05.

Assessing performance in early blind participants on the physical navigation task revealed that both gamers and directed navigators showed similar and high success in navigating the test paths following training with AbES (gamers: 87.5% ±10.4 SD correct, directed navigators: 88.57% ±18.6 SD correct; t = 0.14, p = 0.89) (figure 2 A). Furthermore, mean navigation times were also comparable in both groups (gamers: 75.28 sec ±36.0 SD, directed navigators: 71.34 sec ±73.4 SD; t = 0.13, p = 0.89 ) (figure 2 B).

The similar performance between gamers and directed navigators suggests that both audio-based learning strategies (gamer and directed navigator) allowed for the generation of an accurate spatial representation. In turn, the spatial information acquired through virtual navigation could be transferred to a large-scale, physical navigation task.

### Experiment 2

In the drop off experiment, participants were instructed to exit the building using the shortest path possible from a predetermined starting point. In this experiment, we assessed not only how well spatial information could be transferred, but also mentally manipulated. Given there were a variety of navigation path solutions, routes were scored such that the highest point value was awarded for the shortest possible route (maximum of 3 points) and point values decreased incrementally with path length. Assessing performance revealed that participants in the gamer group showed a distinct advantage over directed navigators in that they were more likely to select the closest exit (i.e. shortest path) regardless of their initial starting point (mean score: 2.50 points ±0.65 SD). In contrast, directed navigators were more likely to use the longest route (mean score: 1.25 points ±0.0 SD) (t = 5.03, p = 0.0002) (fig.2 C). While there was also a trend for faster navigation times in the gamer group, the performance was not significantly different (gamers: 61.4 sec ±52.7 SD, directed navigators: 73.5 sec ±27.1 SD; t = 0.55, p = 0.59).

In contrast to the results of experiment 1, the higher point score observed in the gamer group as compared to the directed navigators suggests that game-based learning strategy may have conferred an advantage in terms of learning the spatial layout of the target building.

## Discussion

We demonstrate that early blind individuals were able to interact with an audio-based virtual environment to generate an accurate spatial cognitive map that corresponds to the spatial layout of a target physical building. The accuracy of this mental map was confirmed by the fact that participants were able to transfer acquired spatial information into successful navigation performance carried out in the physical building modeled in a corresponding virtual environment.

It is of particular interest that participants in the gamer group were able to navigate successfully and at a level of performance comparable to the directed navigation strategy. This is despite the fact that gamers were never explicitly told to retain any information regarding the spatial layout of the building, nor were they aware that they would be assessed on their navigation abilities. This suggests that the interactive and immersive nature of the AbES software and more specifically, the goal directed and exploratory structure inherent to the gaming metaphor, allows for spatial cognitive constructs to be learned easily, accurately, and in an implicit manner. Furthermore, the fact that gamers demonstrated superior performance when asked to find their way out of the building by using the shortest route possible (despite a variety of route possibilities) suggests that the gaming strategy allowed for a more robust and flexible mental manipulation of the spatial information acquired. We interpret these findings as indicative of superior contextual learning and transfer of situational knowledge as a result of a greater understanding regarding the spatial inter-relations within the building environment. In contrast, the directed navigator group tended to use the longest route, regardless of the initial starting point. This strategy is likely more akin to a “constrained functionality” and analogous to rote learning methods that typically fail to capture more global contextual and situational relevant information.

Little is known about the nature and form of spatial cognitive maps in the blind and how they are able to generate these mental representations for the purposes of complex navigation tasks. Recent neuroimaging studies have investigated the neural correlates related to way finding processes and key structures appear to be involved. These include the hippocampus (in tactile maze solving [13]) and occipital cortical areas (in virtual route recognition [14]). Wolbers and colleagues have shown (in sighted subjects) that the parahippocampal place area (PPA) and retrosplenial cortex (RSC) are active in both visual and haptic exploration of complex indoor spatial layouts (i.e. 3D geometrical configurations) [15]. The findings from this study suggest that these areas may be crucial for modality-independent representations of spatial information processing. It would be interesting to determine if similar networks support high-level abstract representations of space in the blind.

The mechanisms related to the transfer of navigation skills from virtual (as well as other orientation aids such as tactile maps) to real environments remain largely unknown. However, the differences in learning strategy (and effects in behavioral performance) observed in this study may be related to the method through which spatial information is characterized and the resultant spatial cognitive map is developed. Within the context of large-scale environments, survey knowledge (i.e. “allocentric” frame) typically describes a more global or holistic “overview” of the surrounding environment. By comparison, route knowledge (i.e. “egocentric” frame) is characterized as a first-person perspective and is typically a precursor to developing survey level knowledge [16]. Flexible route strategies generated from survey level knowledge are key for efficient navigation and way finding, particularly in unfamiliar environments [12]. In the absence of sight, blind individuals are believed to be more reliant on route knowledge despite the fact that developing “higher level” knowledge and spatial skills are considered crucial for promoting greater independence during way finding [10].

In our study, it appears that route level knowledge was obtained through structured and serial learning of the target navigation paths (as evidenced in the directed navigator group), yet there was an apparent lack of survey level knowledge needed for greater success in the drop off task. In contrast, individuals in the gamer group appeared to have a more flexible and robust understanding of their contextual surroundings and possessed a spatial cognitive map that could be manipulated to generate alternate navigation routes. It is possible that acquiring contextual spatial information within a gaming context facilitates a form of visuo-spatial imagery in the blind [17]. This in turn, could allow for the generation of multiple allocentric representations crucial to developing survey level knowledge. Key directions for future research will be to understand how the blind are able to generate accurate and robust spatial information in the absence of sight and how learning through a gaming context facilitates this process.

It is worth acknowledging that the numerous efforts are currently being pursued to improve spatial perception and navigation skills in the blind. These include the development of virtual environments [18], [19] as well as sensory substitution devices (SSDs) [20], [21], [22], [23]. Early results have been promising and design enhancements continue to develop. However, the steep learning curve necessary to develop a high level of proficiency for these devices remains a concern thus limiting the universal adoption of these devices by the blind community. Indeed, it is important that training remains flexible and adaptable so that educational and rehabilitative approaches can be applied to novel and unfamiliar situations and tailored to a person’s particular challenges, needs and learning strategies. Here, the advantage of contextual learning through game play may prove to be a key adjuvant in helping to facilitate this learning process [4], [24].

Finally, studies have compared performance in early blind individuals to normally sighted (blindfolded) controls in spatial navigation tasks. For example (and contrary to previously held views), Fortin and colleagues have shown that blind individuals can outperform their sighted (blindfolded) counterparts in a route learning task [25]. As AbES was specifically designed for the blind community to serve as a potential rehabilitative tool, blindfolded sighted controls were not used in this study. However, the role of previous visual experience on navigation performance remains crucial to our understanding of how spatial mental representations are generated. Ongoing studies will assess performance in late blind individuals in order to address this important issue and keep with the overall goals of developing novel rehabilitative and instructional tools for the blind to promote independence in a society heavily reliant on vision.

## Methods

### Participants

Seventeen early blind participants (all with documented profound blindness acquired prior to the age of 3; see table 1) not previously familiar with the spatial layout of the target building, participated in the study. Participants were randomized to one of two groups; 1) gamers and 2) directed navigators. At no time were the participants informed of the overall purpose of the study nor were they instructed to recall the spatial layout of the building while playing the game. All participants were blindfolded throughout the training and behavioral assessments. Subjects provided signed informed consent prior to participation and the study was approved by the institutional review board of the Massachusetts Eye and Ear Infirmary.

**Table 1:** Study Participants.

Subject	Age	Gender	Cause of Blindness
*Gamer*			
1	19	m	familial exudative vitreo retinopathy
2	24	m	complications due to spinal meningitis
3	30	f	glaucoma
4	41	m	juvenile macular degeneration
5	22	f	retinopathy of prematurity
6	19	m	Peter’s anomaly
7	21	m	retinopathy of prematurity
8	38	f	retinitis pigmentosa
9	19	f	retinopathy of prematurity
*Directed Navigator*			
10	23	f	retinopathy prematurity
11	32	m	cataracts and secondary glaucoma
12	33	m	retinitis pigmentosa
13	44	f	retinitis pigmentosa
14	20	f	lebers congenital amaurosis
15	19	m	glaucoma
16	21	m	optic atrophy
17	29	f	retinitis pigmentosa

### Software

Audio-based Environment Stimulator (AbES) was developed using the XNA programming platform. Based on an original architectural floor plan of an existing building (located at the Carroll Center for the Blind, Newton, MA), a virtual rendering of a modern two-story building was generated. The building includes 23 rooms, a series of connecting corridors, 3 separate entrances and 2 stairwells. As such, the building contains multiple route possibilities to enter and exit. Through an interactive interface that greatly engages a user to actively explore a given environment, auditory-based spatial information is dynamically updated, acquired sequentially, and within context. Each virtual step approximates one step in the real physical building. Wearing stereo headphones and using specific key strokes, a user explores and navigates through the building (moving forward, right or left). Spatial and situational information is based on iconic and spatialized sound cues provided after each step taken (e.g. hearing a knocking sound in the left stereo channel represents the presence of a door on the user’s left side). Orientation is based on cardinal compass headings (e.g. “north”) and text through speech (TTS) is used to provide information regarding a user’s current location, orientation and heading as well as the identity of objects and obstacles in their path. The gaming structure organizes the level into several pre-determined corridors, dead ends, and pathways, giving a sense of the entire area laid out over a three dimensional space. Played out in a corresponding three-dimensional auditory virtual world, the user builds a mental representation of the environment based on these sequential and causal encounters within a goal-directed and exploratory framework (see supplemental video of annotated game play).

### Behavioral Testing and Data Analysis

Both groups interacted with the AbES software for the same amount of time (total of 90 min spread over 3 training sessions). Following training, the participants were taken to the physical building modeled in the AbES software and navigation performance was assessed using two behavioral tasks. In the first set of experiments, navigation accuracy was measured using of a series of predetermined start and finish points. A maximum time of 6 minutes was allowed for each path attempted. The target paths were all of comparable length and complexity (i.e. number of turns). Navigation success (i.e. number of correct paths, expressed as a percent correct) and time to target (seconds) were scored. As a second experiment, a series of “drop off” tasks were carried out in which participants were placed at pre-determined locations and instructed to exit the building (one of three possible exits) using the shortest path possible. Paths were scored such that the shortest path taken was given maximum points (i.e. 3 for the shortest path, 2 for the second, 1 for the longest, and 0 for an incomplete task). Navigation time was also collected. The two behavioral tasks (physical and drop off) were always assessed in the same order. Navigation performance was recorded using a stopwatch carried by an investigator following behind the study participant. Timing commenced once the subject took their first step and stopped when the subject verbally reported that they were in front of the door of the target destination. All data was analyzed using R statistical software. Two participants (one form each group) were excluded from the analysis. T-tests were performed between each group, and we report mean and standard deviation values with significance set at p<0.05.
